# Strain-Specific Infection of Phage AP1 to Rice Bacterial Brown Stripe Pathogen *Acidovorax oryzae*

**DOI:** 10.3390/plants13223182

**Published:** 2024-11-13

**Authors:** Mengju Liu, Yang Zhang, Chunyan Gu, Jinyan Luo, Ying Shen, Xuefang Huang, Xinyan Xu, Temoor Ahmed, Hissah Abdulrahman Alodaini, Ashraf Atef Hatamleh, Yanli Wang, Bin Li

**Affiliations:** 1 State Key Laboratory of Rice Biology and Breeding, Ministry of Agriculture Key Laboratory of Molecular Biology of Crop Pathogens and Insects, Zhejiang Key Laboratory of Biology and Ecological Regulation of Crop Pathogens and Insects, Institute of Biotechnology, Zhejiang University, Hangzhou 310058, China; 11716062@zju.edu.cn (M.L.); taloyament@163.com (Y.Z.); 12216091@zju.edu.cn (X.H.); 12016074@zju.edu.cn (X.X.); temoorahmed@zju.edu.cn (T.A.); 2 Key Laboratory of Plant Genetic Engineering Center of Hebei Province, Institute of Biotechnology and Food Science, Hebei Academy of Agricultural and Forestry Sciences, Shijiazhuang 050070, China; 3 Institute of Plant Protection and Agricultural Product Quality and Safety, Anhui Academy of Agricultural Sciences, Hefei 230001, China; 4 Department of Plant Quarantine, Shanghai Extension and Service Center of Agriculture Technology, Shanghai 201103, China; toyanzi@126.com; 5 Station for the Plant Protection & Quarantine and Control of Agrochemicals of Zhejiang Province, Hangzhou 310004, China; shenying0227@126.com; 6 Department of Life Sciences, Western Caspian University, Baku 1001, Azerbaijan; 7 Department of Botany and Microbiology, College of Science, King Saud University, Riyadh 11451, Saudi Arabia; halodaini@ksu.edu.sa (H.A.A.); ahatamleh@ksu.edu.sa (A.A.H.); 8 State Key Laboratory for Managing Biotic and Chemical Threats to the Quality and Safety of Agro-Products, Institute of Plant Protection and Microbiology, Zhejiang Academy of Agricultural Sciences, Hangzhou 310021, China

**Keywords:** phage, *Acidovorax oryzae*, *Podoviridae*, interaction, LPS

## Abstract

Bacteriophage (phage) AP1 has been reported to effectively lyse *Acidovorax oryzae*, the causative agent of bacterial brown stripe in rice. However, phage AP1 exhibits strain-specific lysis patterns. In order to enhance the potential of phages for biological control of rice bacterial brown stripe, this study investigated the possible mechanism of strain-specific infection by characterizing phage AP1 and its susceptible (RS-2) and resistant (RS-1) strains. Based on the current classification standards and available database information, phage AP1 was classified into the class *Caudoviricetes*, and it is a kind of podophage. Comparative analysis of the susceptible and resistant strains showed no significant differences in growth kinetics, motility, biofilm formation, or effector Hcp production. Interestingly, the resistant strain demonstrated enhanced virulence compared to the susceptible strain. Prokaryotic expression studies indicated that six putative structural proteins of phage AP1 exhibited varying degrees of binding affinity (1.90–9.15%) to lipopolysaccharide (LPS). However, pull-down assays and bacterial two-hybrid analyses revealed that only gp66 can interact with four host proteins, which were identified as glycosyltransferase, RcnB, ClpB, and ImpB through immunoprecipitation and mass spectrometry analyses. The role of LPS in the specific infection mechanism of phage AP1 was further elucidated through the construction of knockout mutant strains and complementary strains targeting a unique gene cluster (*wbzB*, *wbzC*, *wbzE*, and *wbzF*) involved in LPS precursor biosynthesis. These findings provide novel insights into the mechanisms of phage-host specificity, which are crucial for the effective application of phage AP1 in controlling rice bacterial brown stripe.

## 1. Introduction

Rice bacterial brown stripe is caused by the Gram-negative bacterium *Acidovorax oryzae* (formerly classified as *Acidovorax avenae* subsp. *avenae*), which has been reported to exhibit strong virulence towards rice [1,2,3,4,5]. Moreover, *Acidovorax* sp. have demonstrated resistance to *β*-lactam antibiotics, suggesting that a range of conventional pesticides may be ineffective against these pathogens [6]. Therefore, there is an urgent need for alternative, environmentally sustainable, and efficient methods to control this disease. Indeed, numerous beneficial microorganisms with antagonistic activity against this rice bacterial pathogen have been isolated and characterized. Concurrently, increasing attention has been focused on bacteriophages (phages), which represent the most abundant biological entities in the biosphere. Current estimates suggest a global phage population of approximately 10^31^ particles [7].

The first step of phage infection is adsorption to the surface of host bacteria by specifically binding host recognition proteins or receptor binding proteins (RBPs) to the receptors. RBPs can recognize cell surface substances, including proteins, carbohydrates, teichoic acids, and O-antigen [8,9]. For example, the first identified RBP in Gram-positive lactic acid bacteria phages adsorbs and infects the host by using surface proteins as receptors [10]. Subsequently, the identified RBPs of phages TP901-1 and Tuc2009 specifically recognize carbohydrate portions on the surface of host *Lactococcus lactis* [11]. Furthermore, it is more complex for RBPs of Gram-negative bacteria phages, which often utilize tailspike proteins as RBPs, to bind to the O-antigen receptors of the host [12,13,14,15,16,17,18].

Lipopolysaccharides (LPSs) are complex macromolecules located on the outermost layer of Gram-negative bacterial cell envelopes. They consist of three main components: the O-antigen, core oligosaccharide, and lipid A [19,20]. The synthesis of LPSs consists of two parts: the lipid A-core oligosaccharide portion and the O-polysaccharide synthesis portion. The synthesis of the latter involves three pathways [21]; two are particularly well-characterized: the Wzy-dependent polymerization pathway and the ABC transporter-dependent synthesis pathway. LPSs have been found to be associated with the virulence of plant pathogenic bacteria *Erwinia amylovora*, *Ralstonia solanacearum*, *Xanthomonas axonopodis* pv. *Citri*, and *Xylella fastidiosa* [22,23,24,25]. Moreover, LPSs serve as the primary receptor for various bacteriophage morphotypes, including those with long tails, short tails, and contractile tails.

In recent years, bacteriophages have gained attention as potential biocontrol agents for plant bacterial diseases. However, a significant challenge in phage therapy is the emergence of phage-resistant pathogens. This resistance is crucial for bacterial survival and adaptation, and it directly impacts the development of effective phage therapies against resistant pathogens [26]. One of the main mechanisms of phage resistance is the mutation of receptors, which not only prevents phage attachment by altering the surface molecules that phages recognize [27] but also triggers coevolutionary dynamics between phages and bacteria, leading to the diversification and adaptation of both populations [28,29]. Additionally, receptor mutation can also have pleiotropic effects on bacterial fitness and virulence, depending on the phage–host system and the environmental conditions [30].

Phage AP1 of the circle genome belongs to the class *Caudoviricetes*, comprising 55,676 bp with 87 open reading frames, with a 70 nm diameter icosahedral head and a 45 nm non-contractile tail, based on the analysis of genome sequences and restriction endonuclease as well as observation of transmission electron microscopy. Our study reported that bacteriophage AP1 can effectively lyse *A. oryzae* through a novel holin–endolysin lysis cassette, suggesting its potential for controlling this rice bacterial disease. However, some strains of *A. oryzae* exhibited resistance to phage AP1 infection [31]. The objective of the present study is to provide a more comprehensive understanding of the specific interactions between bacteriophage AP1 and its host, *A. oryzae*, with a particular focus on the mechanisms underlying phage resistance. Additionally, we characterized the morphological and molecular features of both resistant and susceptible bacterial strains, as well as their interactions with phage AP1, in order to enhance the potential application of phage therapy for controlling rice bacterial brown stripe disease.

## 2. Results

### 2.1. Strain-Specific Infection of Phage

**Figure 1:**
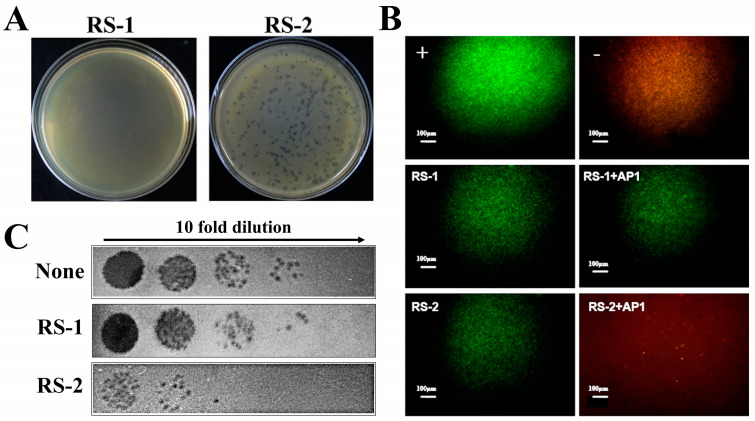
The discovery of differential infection. (**A**): Sensitivity of phage AP1 to strains RS-1 and RS-2. (**B**): Fluorescence staining assay for detecting bacterial sensitivity to phage; “+” represents the positive control, which is live bacteria without the addition of the phage, appearing green under fluorescence. “−” represents the negative control, where the addition of isopropanol kills the bacteria, appearing red under fluorescence. (**C**): The phage adsorption difference between strains RS-1 and RS-2. “None” represents the use of the phage that has not been incubated with any bacterial culture. “RS-1” represents the supernatant obtained after incubating the phage with a culture of strain RS-1 for 10 min and then centrifuging. “RS-2” represents the supernatant obtained after incubating the phage with a culture of strain RS-2 for 10 min and then centrifuging. The titration strain used is RS-2. All the supernatants were diluted in a 10-fold gradient before use.

The sensitivity of strains RS-1 and RS-2 to phage AP1 was detected using the double-layer agar method. As shown in Figure 1A, there is a difference in phage sensitivity between strains RS-1 and RS-2. Indeed, clear plaques were observed for strain RS-2, but no plaques appeared for strain RS-1. Similar experimental results were also observed in bacterial viability tests using fluorescence microscopy (Figure 1B). The overnight bacterial culture without the phage showed green fluorescence as a positive control, indicating live cells, while the overnight bacterial culture damaged by isopropanol showed red fluorescence as a negative control, indicating dead cells. Furthermore, green fluorescences were observed in strains RS-1 and RS-2 alone, representing live cells for the two bacterial strains. After adding the phage to strain RS-1, there was no obvious change in the color of bacterial cells, indicating that phage AP1 was unable to lyse and kill strain RS-1. However, red fluorescences were clearly found when adding the phage AP1 to strain RS-2, indicating that many bacterial cells were destroyed or even killed. Therefore, it can be inferred that the activity of strain RS-2 was affected by phage AP1, with most of the bacterial cells lysed and killed.

### 2.2. Receptors Contribute to Differential Infection

Adsorption experiments were used in this study to reveal the role of receptors in differential infection between strains RS-1 and RS-2. As shown in Figure 1C, there was a great difference between strains RS-1 and RS-2 in the number of remaining phages in the bacterial supernatant, which was determined based on the spot assay by incubating phage AP1 with strains RS-1 and RS-2, removing bacterial cells by centrifugation, and then spotting tenfold serially diluted supernatants on plates. Indeed, the amount of remaining phage in strain RS-1 is 10^7^ PFU/mL, which is almost the same as that in the negative control; however, compared to strain RS-1, strain RS-2 shows a nearly two orders of magnitude reduction in the amount of remaining phage, with only 10^5^ PFU/mL. This indicates that phage AP1 was able to be greatly adsorbed by strain RS-2 but was unable to be adsorbed by strain RS-1. Therefore, it can be inferred that the adsorption may be involved in the difference between strains RS-1 and RS-2 in AP1 sensitivity, highlighting the importance of receptors such as LPS in phage infection of bacteria.

### 2.3. Biological Differences Between Phage-Sensitive and Resistant Strains

**Figure 2:**
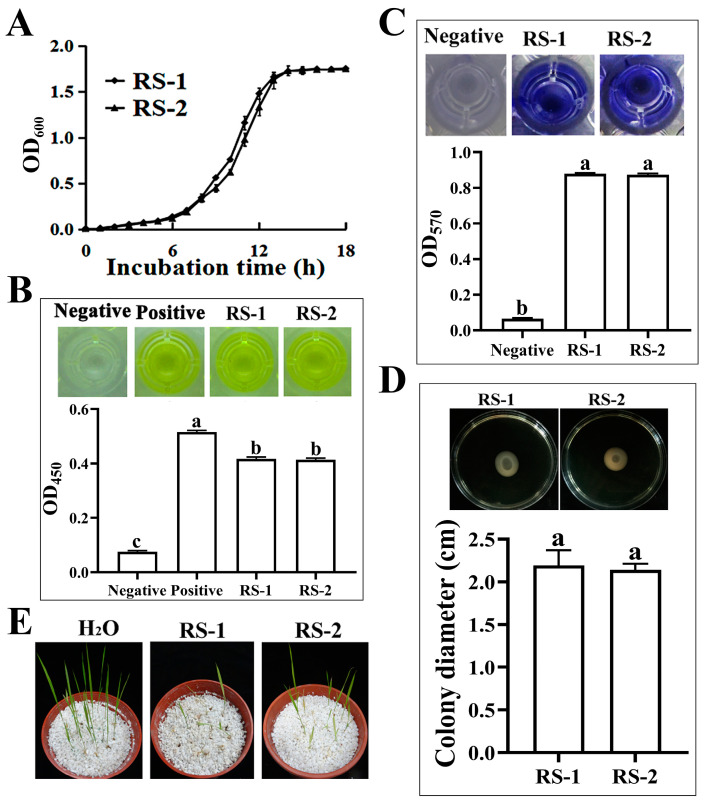
Biological character comparisons of strains RS-1 and RS-2. (**A**): Bacterial growth. (**B**): ELISA of Hcp; negative represents purified His protein, and positive represents purified Hcp-His fusion protein. (**C**): Biofilm formation; negative represents LB medium without any bacterial culture added. (**D**): Motility. (**E**): Pathogenicity. Columns with different letters are significantly different according to the LSD test (*p* = 0.05).

The intrinsic reasons behind the differences in phage infection between strains RS-1 and RS-2 were further determined by comparing bacteriological traits, including cell growth, secretion of type VI secretion system effector protein Hcp, biofilm formation, pathogenicity to rice seedlings, and bacterial motility. The results showed that the two strains exhibited a high degree of similarity in these phenotypes except for the pathogenicity to rice. Indeed, the result of growth curves indicated that there was no significant difference in the OD_600_ values between the two strains at 18 different time points, while the OD_600_ values of strains RS-1 and RS-2 were 1.725 and 1.731, respectively, after 14 h of incubation. These results showed that the two strains have a similar growth rate and growth capacity (Figure 2A). The secretion of Hcp protein was determined by the ELISA detection assay based on the appearance of a yellow color in the filtered suspensions of strains RS-1 and RS-2, which is similar to the positive control. Furthermore, the ELISA results revealed that there were no significant differences in the Hcp secretion between the two strains, while the OD_450_ values of strains RS-1 and RS-2 were 0.416 and 0.413, respectively (Figure 2B).

Similarly, no significant differences between strains RS-1 and RS-2 were observed in biofilm formation (Figure 2C) or bacterial motility (Figure 2D). Biofilm formation was determined based on the method of crystal violet staining by measuring the OD_570_ value after 48 h of incubation. The mean OD_570_ values of strains RS-1 and RS-2 were 0.880 and 0.875, respectively. Bacterial motility was determined based on the motility assay; the colony diameters of strains RS-1 and RS-2 were 2.19 cm and 2.14 cm, respectively, after 48 h of incubation. Furthermore, pathogenicity was determined by measuring the height of rice seedlings after 10 d of inoculation. As shown in Figure 2E, the rice treated with the water control showed healthy and normal growth, with an average plant height of 8.83 cm. In contrast, rice treated with strains RS-1 and RS-2 exhibited shorter and weaker leaves, with average plant heights of only 3.80 cm and 1.71 cm, respectively. Both strains RS-1 and RS-2 significantly inhibited the germination and growth of rice seedlings; however, the former exhibited a 23.41% greater reduction in plant height than that of the latter. Thus, these results showed that strains RS-1 and RS-2 are highly similar, with the most notable differences being in their pathogenicity to rice seedlings and sensitivity to phage AP1.

### 2.4. LPS Can Bind with Structural Proteins of Phage AP1

**Figure 3:**
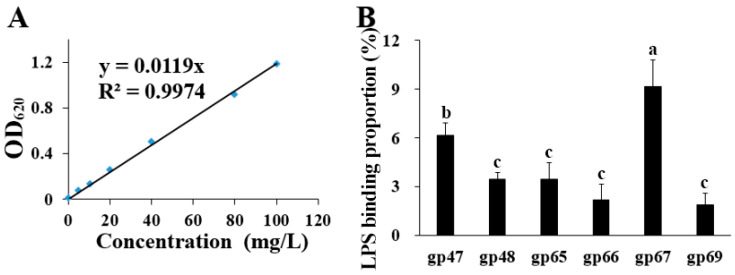
Identification of putative structural proteins in phage AP1 with binding ability to bacterial LPS. (**A**): LPS concentration determination standard curve. (**B**): Binding proportion to the host LPS of phage AP1 putative structural protein. The amount of LPS adsorbed by each protein divided by the initial amount of LPS added represents each binding proportion to LPS. Columns with different letters are significantly different according to the LSD test (*p* = 0.05).

Host recognition proteins of phage AP1 were determined based on their interaction with bacterial phage receptors such as LPS, which was primarily conducted by identifying the putative phage structural proteins gp47, gp48, gp65, gp66, gp67, and gp69 with the binding ability to the LPS receptor. The standard curve was generated by plotting known concentrations of LPS on the x-axis against the corresponding absorbance readings on the y-axis. A linear regression line was fit to the data (R^2^ = 0.9974), providing the equation y = 0.119× for concentration calculation. The standard curve was reliable within a range of 0 to 100 mg/L. This strong linear relationship indicates that the equation can be used to calculate the binding proportion to LPS of the phage proteins (Figure 3A). The binding experiment showed that all six putative phage structural proteins exhibited the ability to bind to LPS, and the binding proportions to the LPS of gp47, gp48, gp65, gp66, gp67, and gp69 were 6.20%, 3.46%, 3.47%, 2.20%, 9.15%, and 1.90%, respectively (Figure 3B). Phage protein gp67 had the highest binding proportion to LPS, with gp47 being the second highest, although several other phage proteins also show a small amount of binding. This indicates that gp67 and gp47 have a more significant binding ability to LPS compared to other proteins. This result suggests that phage AP1 infects strain RS-2 by binding to the receptor LPS of host bacteria.

### 2.5. Host Proteins Interacting with Phage AP1 Structural Proteins

**Figure 4:**
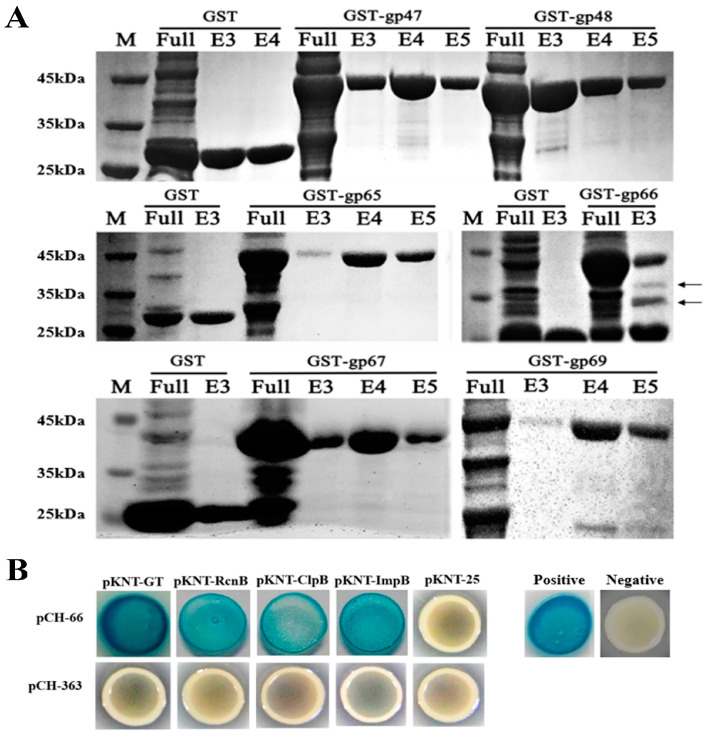
Interaction between host recognition protein of phage AP1 and host protein. (**A**): Proteins from the host can be co-eluted by gp66. (**B**): Identification of protein–protein interactions by B2H.

A receptor of phage AP1 was identified by using in vitro expressed phage structural proteins as baits to capture proteins from host bacterium RS-2 that interact with them. The results showed that most bait proteins did not co-elute with proteins of the host bacterium, but bait protein gp66 was found to co-elute with several host proteins (Figure 4A). This reveals the interaction between bait protein gp66 and these co-eluted proteins from strain RS-2. As described in Table 1, the following four host proteins were identified as potential receptors by excising the differential bands of bait protein gp66 and sending them for mass spectrometry analysis. Glycosyltransferase encoded by gene 2086 shows the highest abundance, with a matched peptide count of seven. Transmembrane protein RcnB encoded by gene 1561 consists of six matched peptides. The other two proteins ClpB and ImpB belong to the component of the Type VI secretion system, each with three matched peptides. Furthermore, the interaction between these four host proteins from strain RS-2 and bait protein gp66 of phage AP1 was verified by using a bacterial two-hybrid blue–white screening assay, which was carried out by incubating proteins in the dark at 23 °C for 48 h, the results showed that the four proteins did not interact with the corresponding empty plasmids, as the colonies remained white; however, all four proteins can interact with gp66, as evidenced by the colonies turning blue (Figure 4B).

### 2.6. Genes Associated with Strain-Specific Infection of Phage

**Figure 5:**
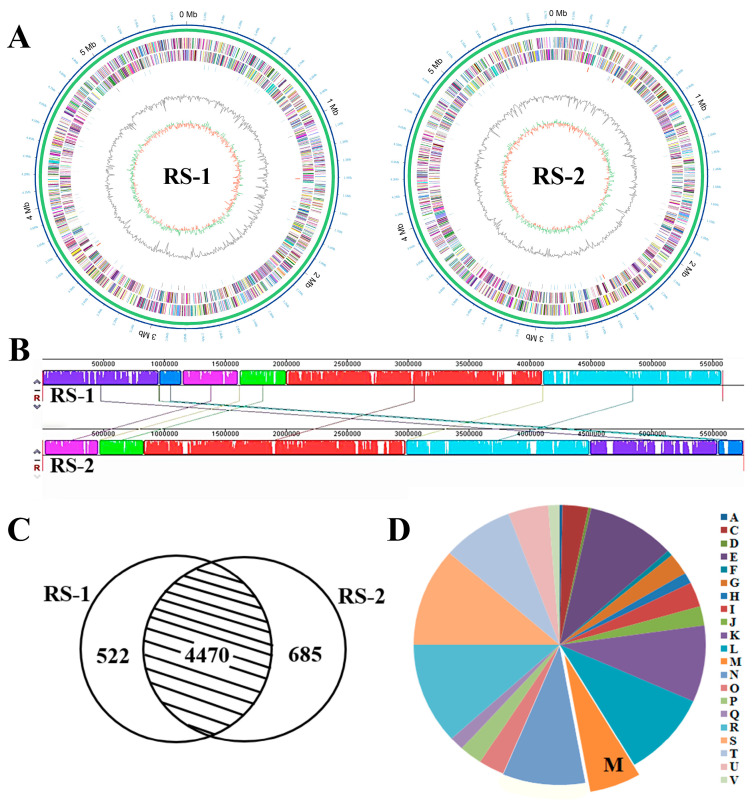
Genomic comparisons between AP1-resistant strain RS-1 and sensitive strain RS-2. (**A**): Whole genome profiles. (**B**): Genome alignment by Mauve. (**C**): Number of differential genes. (**D**): COG classification of unique genes in RS-2 (A: RNA processing and modification. C: Energy production and conversion. D: Cell cycle control, cell division, and chromosome partitioning. E: Amino acid transport and metabolism. F: Nucleotide transport and metabolism. G: Carbohydrate transport and metabolism. H: Coenzyme transport and metabolism. I: Lipid transport and metabolism. J: Translation, ribosomal structure, and biogenesis. K: Transcription. L: Replication, recombination, and repair. M: Cell wall/membrane/envelope biogenesis. N: Cell motility. O: Post-translational modification, protein turnover, and chaperones. P: Inorganic ion transport and metabolism. Q: Secondary metabolites biosynthesis, transport, and catabolism. R: General function prediction only. S: Function unknown. T: Signal transduction mechanisms. U: Intracellular trafficking, secretion, and vesicular transport. V: Defense mechanisms).

To identify genes associated with the receptor of phage AP1, genomes of phage-resistant strain RS-1 and sensitive strain RS-2 were sequenced by third-generation sequencing technology, and data were released on NCBI (Accession no. CP169238 and CP169239). Figure 5A shows the whole genome information of RS-1 and RS-2. In general, the genome sizes of strains RS-1 and RS-2 are approximately 5 Mb (Figure 5A), while analysis with Mauve software revealed a high similarity in both genomes of the two strains with 87.4% of homologous regions and 97.9% of ANI (Average Nucleotide Identity) values (Figure 5B). A total of 4470 genes were identified as homologous genes, while there were 522 and 685 unique genes in strains RS-1 and RS-2, respectively (Figure 5C). In particular, 5.7% of unique genes in strain RS-2 were annotated as biosynthesis of cell membrane/cell wall (Figure 5D). The detailed genomic information of strains RS-1 and RS-2 are displayed in Table 2. The GC content in RS-1 and RS-2 was 68.64% and 68.60%, respectively. The number of RNA genes in RS-1 and RS-2 was 78 and 75, respectively.

**Figure 6:**
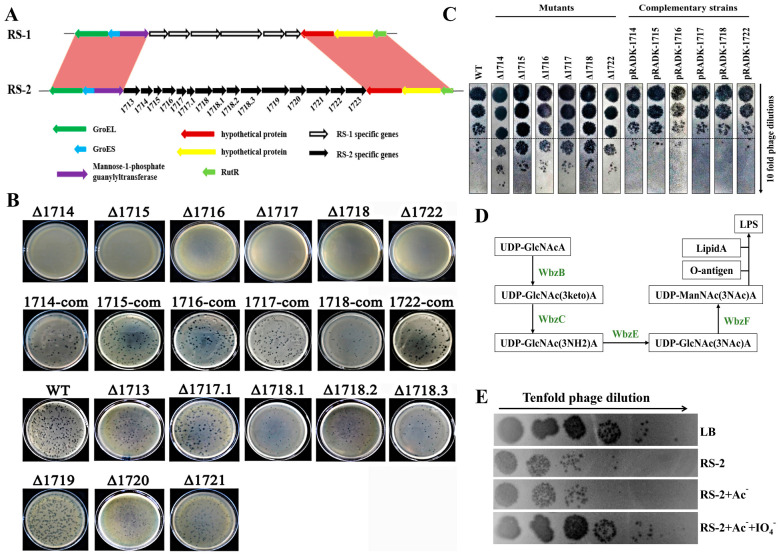
LPS receptor identification of phage AP1. (**A**): Comparative analysis of the unique gene clusters in *Acidovorax oryzae* RS-1 and RS-2; (**B**): Sensitivity of phage AP1 to host mutant strains with mutation of functional genes related to the biosynthesis of the cell membrane/cell wall. (**C**): Residual phage amount after adsorption. (**D**): Unique gene clusters in RS-2 are involved in the synthesis of LPS precursor. (**E**): Confirmation that LPS is the receptor for phage AP1 infecting strain RS-2 using periodic acid. Ac^−^: acetate buffer; IO_4_^−^: periodic acid.

Comparative genome analysis of both strains identified a unique gene cluster involved in the biosynthesis of the bacterial cell wall in strain RS-2 (Figure 6A). Fifteen genes were included in this unique gene cluster of strain RS-2, which was designated as *orf1714*-*1723*. These genes exhibited a differential effect in the interaction of strain RS-2 with phage AP1. Indeed, the double agar overlay plaque assay showed that mutants of six unique genes (*orf1714*, *orf1715*, *orf1716*, *orf1717*, *orf1718*, and *orf1722*) were found to be resistant to the infection of phage AP1, and their sensitivity to phage AP1 was restored after gene complement, while there was no difference in phage infection between the wild type and mutants of the other nine unique genes (Figure 6B).

Compared to the wild type, the mutation of six unique genes (*orf1714*, *orf1715*, *orf1716*, *orf1717*, *orf1718*, and *orf1722*) significantly reduced the adsorption of phage AP1 to strain RS-2, indicating that these genes may be functionally related to phage receptor (Figure 6C). Indeed, these mutants caused a nearly an order of magnitude reduction in the number of remaining phages compared to the wild type (10^8^ PFU/mL), emphasizing their important role in the infection of phage AP1 to strain RS-2. Furthermore, WbcB, WbcE, WbcC, and WbcF, encoded by *orf1714*-*1717*, respectively, were found to be associated with the synthesis of the LPS precursor UDP-ManNAc (3NAc) A (Figure 6D). Therefore, it can be inferred that LPS plays a crucial role in the infection of strain RS-2 by phage AP1, which may also be the main cause of why it was unable to infect strain RS-1.

The receptor of phage AP1 was further confirmed by disrupting the surface LPS on the bacterial cells of strain RS-2 using periodic acid. As shown in Figure 6E, the periodic acid-treated bacteria almost completely lost their ability to adsorb phage AP1. Indeed, the number of remaining phages after incubation with the periodic acid-treated bacteria was nearly 10^8^ PFU/mL, which is the same as the number of phages in the group without added bacteria but was approximately 100 times higher than that in the two control groups (bacteria treated with acetate buffer and water since periodic acid is dissolved in acetate buffer). Therefore, it can be concluded that LPS was the receptor of phage AP1 infecting strain RS-2.

## 3. Discussion

Phage therapy offers a promising solution for the control of rice bacterial diseases. In this study, we found that phage AP1 exhibits strain specificity during infection. Similar situations frequently occur in the infection processes of other phages as well. For example, a total of 20,000 phage–host combinations were analyzed, revealing significant differences in the phage profiles of *P. syringae* pv. *actinidiae* strains (Psa) from different geographic regions. For instance, phage Psa259 specifically infects the Japanese ICMP 9853 strain of Psa, while phage Psa271 targets only the New Zealand strains of Psa [32]. Gill et al. [33] divided 50 *Erwinia amylovora* phages into six groups by testing their host ranges. Phages in groups 1, 2, 4, and 5 were able to form plaques on 11 or more of the 13 tested *E. amylovora* strains, whereas phages in group 3C (phage PEa1) and group 3B showed little to no visible lytic activity against *E. amylovora* strains Ea29-7, BC29, Ea34A, BC34A, and BC1280, as well as against bacterial strains EaG-5 and Ea6-4 isolated from Harrow, Ontario. Bhunchoth et al. [34] isolated 14 phages infecting *Ralstonia solanacearum* from soil samples collected in Chiang Mai, Thailand. When tested against 59 *R. solanacearum* strains isolated from Thailand and Japan, these phages also exhibited different host ranges.

Phage infection specificity refers to the high selectivity that phages exhibit when infecting different bacterial hosts. Different phages typically can only infect specific bacterial species or strains, and this specificity depends on the interaction between the phage and bacterial surface receptors [35]. Phage T4 specifically infects some particular strains of *Escherichia coli*. This specificity is partly due to the interaction between the tail fiber proteins of phage T4 and the outer membrane receptors of *E. coli* [36]. Phage λ specifically infects certain strains of *E. coli* by binding to the maltose-binding protein receptor on their outer membrane. This specificity makes phage λ an important model for studying phage–host interactions [37]. A recent comprehensive systematic study showed that *Enquatrovirus* phages share exactly the same range of lysing hosts due to their common recognition of conserved receptors using homologous receptor binding proteins [38]. Phage P22 is a phage that specifically infects *Salmonella enterica* by recognizing and binding to the O-antigen on the bacterial surface [12]. These examples demonstrate how phages infect by specifically recognizing particular receptors on the surface of their host bacteria, which is consistent with our result. The diversity of these receptors contributes to the host specificity of phage infections. By understanding the infection specificity of phages, it is possible to more effectively select and design phages for the control of specific pathogenic bacteria [39].

The initiation of phage infection is triggered by the specific recognition between the receptor-binding proteins (RBPs) located at the tip of the phage tail and the receptors on the surface of the host cell. This specificity is directly related to the specificity of adsorption and is associated with the structure of the receptors on the host cell surface [9,40]. In Gram-negative bacteria, these receptors are mainly composed of LPSs, outer membrane proteins (OMPs), and components of fimbriae or capsules [41]. Recent research on the receptors used by phages to infect *Escherichia coli* and related gut bacteria has revealed that *Siphoviruses* typically target porin proteins on the bacterial surface, *Podoviridae* phages are known to target polysaccharides, and *Myoviridae* phages can target either porin proteins or polysaccharides [42]. These variations in bacterial cell wall composition, thickness, lipid and lipoprotein content, and receptor specificity indicate that phages have evolved to infect particular bacterial hosts. This underscores the significance of understanding the molecular mechanisms involved in phage–host interactions.

Receptor recognition is a highly specific process and is part of the natural mechanism of host specificity. In Gram-negative bacteria, outer membrane proteins or LPS may serve as specific phage receptors [43]. LPS has been widely reported to be the receptor of phages [30,44,45,46]. In this study, we identified the LPS of *A. oryzae* strain RS-2 as the receptor for phage AP1. We identified that the synthesis of the phage AP1 receptor is related to *wbzB*, *wbzC*, *wbzE*, and *wbzF*, belonging to the wb* gene cluster, which has been reported in *Vibrio cholerae* serogroup O1 and was responsible for the O-antigen biosynthesis of phage VP4 receptor [47]. Orthologous genes were also found to be involved in the synthesis of the LPS precursor in *Pseudomonas aeruginosa* and *Bordetella pertussis* [48,49,50]. Therefore, the difference in the O-antigen precursor synthesized by the wb* gene is one of the important reasons for the difference in the adsorption ability of phage AP1 to strains RS-1 and RS-2.

During the search for the phage AP1 receptor, we discovered an RS-2-specific gene cluster associated with OPS (O-specific polysaccharide) synthesis and transport. Furthermore, it is well known that OPS is a part of bacterial LPS located on the outer membrane of bacteria, which can interact with the host recognition system and provide specific antigenicity on the bacterial surface. Four genes within this cluster, *wbzB*, *wbzC*, *wbzE*, and *wbzF*, collectively catalyze the synthesis of UDP-ManNAc3NAcA (Uridine diphosphate N-acetylmannosamine 3-N-acetyl-amino-3-deoxy-α-D-mannuronic acid), a crucial intermediate in the synthesis of bacterial extracellular polysaccharides, such as the O-antigen of Gram-negative bacteria lipopolysaccharides [48,50]. While OPS transfer systems in these bacteria typically rely on the Wzy polymerase system, analysis of this unique RS-2 gene cluster revealed that it is dependent on an ABC transporter system for synthesis and transport. A mutation in the OPS transport protein wzt (*orf1718*) resulted in phage resistance, further suggesting that this gene cluster plays a key role in the recognition and infection of RS-2 by phage AP1. Additionally, within this unique gene cluster, a glycosyltransferase (*orf1722*) associated with phage AP1 infection was identified. Conserved structural analysis showed that this glycosyltransferase belongs to the GTB protein family, with a characteristic Rossmann fold at both the N-terminal and C-terminal [51,52].

In the strain-specific gene cluster of RS-2, there are a total of 15 genes. Besides the six genes that are associated with phage infection, the remaining nine genes do not play a direct role in the phage infection process. The aforementioned protein wzt (*orf1718*), as an ABC transporter-related protein, affects bacterial resistance to phages. Protein wzm (*orf1717.1*) encoding genes are also associated with ABC transporters; however, it does not affect the infection of phage AP1. A similar situation occurs with orf1718.2, orf1718.3, orf1720, and orf1723. Like orf1722, these four proteins are also classified as glycosyltransferases, but their deletion does not completely affect the infection of phage AP1. This could be due to their different substrate specificities. Different glycosyltransferases may recognize different substrate molecules, even though they all perform glycosylation reactions. The exact reason for this remains unclear, which may illustrate the complexity of gene functions. As for orf1718.1, analysis has revealed that it contains a methyltransferase domain, which may add a methyl group to the end of the OPS (O-polysaccharide) chain, thereby terminating further OPS chain synthesis. Additionally, a coiled-coil structure is present at its C-terminus, resembling the structure of glycan chain terminase reported in the literature. Therefore, it is preliminarily determined that orf1718.1 is related to the termination of OPS chain synthesis. This might be why its mutation does not fully affect the infection of phage AP1. Both orf1719 and orf1721 are asparagine synthetases, which may be involved in the amination of sugars. A domain highly homologous to asnB was found in orf1719. However, there is currently no research indicating that asnB and other amidases are involved in the OPS (O-polysaccharide) synthesis process.

In this study, four host proteins were identified to interact with the phage structural protein gp66, with the most abundant being glycosyltransferase. Glycosyltransferases are enzymes responsible for transferring glycosyl groups from active donor molecules (e.g., UDP-sugar) to acceptor molecules, playing a critical role in the biosynthesis of polysaccharides, glycolipids, and glycoproteins [53]. Interestingly, glycosyltransferase has been reported to play an important role in the transfer of UDP-ManNAc3NacA. The unusual sugar 2,3-diacetamido-2,3-dideoxy-d-mannuronic acid (ManNAc3NacA) has been observed in the lipopolysaccharides of both pathogenic and nonpathogenic Gram-negative bacteria. It is added to the LPS of these organisms by glycosyltransferases that use UDP-ManNAc3NacA as substrates [54].

In agreement with the experiment, specific infection is highly associated with gene clusters (*wbzB*, *wbzC*, *wbzE*, and *wbzF*) encoding UDP-ManNAc3NAcA, which is transferred to its corresponding receptor molecule by glycosyltransferase, thereby participating in the construction of bacterial cell walls and LPSs. Specifically, in certain bacteria, specific glycosyltransferases can recognize UDP-ManNAc3NAcA and integrate it into complex polysaccharide structures, a process essential for bacterial growth and immune evasion.

There is a conflicting result in the interact test of structural proteins gp47, gp48, gp65, gp66, gp67, and gp69 of phage AP1 with host bacteria. For example, structural protein gp66 of phage AP1 did not show prominent binding with the LPS of the host bacterium in the LPS binding experiments. However, only gp66 out of the six structural proteins interacted with the total protein of strain RS-2 in the co-elution experiments. The inconsistency may be mainly due to the difference in the component of the host bacterium, with LPS in the former and cells in the latter.

It was observed that the four proteins interacting with gp66 play important roles in the transmembrane process. Therefore, we hypothesize that phage AP1 may initially attach to the bacterial surface via the six putative structural proteins tested in this study, particularly gp47 and gp67. Following this attachment, gp66 likely interacts with bacterial transmembrane-related proteins to facilitate further entry into the host bacterium. Studies have indicated that after the initial binding of the phage to the receptor, a transmembrane channel must form to cross both membranes and the periplasmic space, facilitating efficient DNA transfer [55].

Strains RS-1 and RS-2 were specifically chosen because they represent contrasting phenotypes in phage sensitivity while maintaining similar growth characteristics. As demonstrated in our results (Figure 2), RS-1 and RS-2 show remarkably similar traits in terms of growth kinetics, biofilm formation, motility, and Type VI secretion system effector (Hcp) production. This similarity in other characteristics makes them ideal model strains for studying phage resistance mechanisms, as it reduces confounding variables that could affect phage sensitivity. The identification of LPS as a key factor in phage sensitivity provides a molecular marker that can be used to predict phage susceptibility in other strains. The *wbz* gene cluster (*wbzB*, *wbzC*, *wbzE*, and *wbzF*) involved in LPS precursor biosynthesis represents a conserved mechanism that can be used to screen other strains for potential phage sensitivity.

## 4. Materials and Methods

### 4.1. Bacteria, Phage, and Plasmids

Strains RS-1 and RS-2 of *A. oryzae* used in this study were previously isolated from diseased rice plants in Zhejiang, China, and stored in the Institute of Biotechnology, Zhejiang University, Hangzhou, China [56]. Phage AP1 was isolated from diseased rice seeds caused by *A. oryzae* in Zhejiang, China, using a double agar overlay plaque assay, while strain RS-2 was used as the indicator strain [31]. Luria–Bertani (LB) medium (10 g tryptone, 5 g yeast extract, and 10 g NaCl per liter) was used to grow the bacterial strains and to propagate the phage. *A. oryzae* strains were cultured at 30 °C, while *Escherichia coli* strains were grown at 37 °C. Details of the bacterium and plasmids used in this study are provided in Table 3.

### 4.2. Infection of Phage to Host Bacteria

#### 4.2.1. Double-Layer Agar Assay

The infection of the phage to strains RS-1 and RS-2 of *A. oryzae* was determined by using a double-layer agar assay, which was carried out as described by Ogunyemi et al. [59]. In brief, a single bacterial colony was picked up and inoculated into 5 mL LB. After overnight incubation at 30 °C with shaking at 200 rpm/min, 100 μL of bacterial culture with 0.6 of OD_600_ and 50 μL of phage (10^2^ PFU/mL) were added to 5.0 mL LB (0.7% agar) medium and then overlaid on LB (1.5% agar) medium. The double plates were incubated at 30 °C for 24 h for the observation of plaques. Each treatment was performed in triplicate, and the experiment was repeated three times.

#### 4.2.2. Fluorescence Staining Assay

The lysing and killing effect of phage AP1 on strains RS-1 and RS-2 of *A. oryzae* was determined based on the fluorescence staining assay, with 10 μL of phage AP1 (10^7^ PFU/mL) being mixed with 1 mL overnight bacterial culture, and then incubated at 30 °C for 4 h, which was carried out as described by Springer et al. [60] using GeneCopoeia’s cell fluorescent dyes. The overnight bacterial culture without the phage was used as positive control and the overnight bacterial culture damaged by isopropanol was used as negative control. Indeed, nucView green stains live cells green, while propidium iodide stains dead cells red. Thus, live and dead bacterial cells can be distinguished under a fluorescence microscope (Zeiss Axio Imager A2, Oberkochen, Germany) by observing the color of stained cells.

#### 4.2.3. Phage Adsorption Efficiency Assay

The phage adsorption efficiency of the mutant and complement strains was determined by the double-layer agar assay, which was carried out as previously reported [61]. In brief, 1 mL of overnight bacterial culture with OD_600_ = 1.0 was mixed with 100 μL of phage AP1(10^5^ PFU/mL) in an LB broth medium with antibiotics if necessary (50 μg/mL kanamycin or 10 μg/mL chloromycetin), incubated at room temperature for 10 min, and then centrifuged at 13,000× *g* for 5 min. The supernatant was removed, and a 10-fold dilution gradient was performed. Then, 100 μL of bacterial culture with 0.6 OD_600_ was added to 5.0 mL LB (0.7% agar) medium and then overlaid on LB (1.5% agar) medium. After solidification, the plates were spotted using 2 μL of gradient-diluted phage sequentially and then incubated at 30 °C for 24 h for the observation of plaques. Each treatment was performed in triplicate and the experiment was repeated three times.

### 4.3. Characterization of Resistant/Susceptible Strains

#### 4.3.1. Bacterial Growth Assays

The bacterial growth of strains RS-1 and RS-2 was determined as described by Luo et al. [62] by measuring the OD_600_ of bacterial cultures at different incubation times. In brief, 1 mL of overnight bacterial cultures with an OD_600_ of 0.6 was inoculated into 100 mL of fresh liquid LB medium and then shaken at 200 rpm/min at 30 °C. The OD_600_ of bacterial cultures was measured at the specified time points. Each treatment had three replicates, and the experiment was repeated twice. The growth curves of bacteria were generated by plotting time on the x-axis and the mean OD_600_ on the y-axis.

#### 4.3.2. Biofilm Formation Assay

The biofilm formation of strains RS-1 and RS-2 was determined based on the crystal violet method described by Coenye et al. [63], which was carried out by inoculating each well of a 96-well plate with 100 μL overnight bacterial cultures with (OD_600_ = 0.5) and then incubating statically at 30 °C for 48 h. To each well of the plate, crystal violet solution was added after gently pouring off the bacterial suspension; then, it was rinsed twice with ddH_2_O, air-dried naturally, and then incubated at room temperature for 30 min. The absorbance values of each well at 570 nm were measured using a microplate reader (ThermoFisher Scientific Inc., Waltham, MA, USA) after gently discarding the unbound crystal violet solution, rinsing 2–3 times with ddH_2_O, and air drying naturally. Each treatment had 12 replicates, and the experiment was performed independently three times.

#### 4.3.3. Motility Assay

The motility assay was carried out based on the method of Bahar et al. [64] with some modifications. In brief, single colonies were picked up and inoculated into an LB liquid medium. After shaking overnight (200 rpm/min) at 30 °C, 5 μL of the bacterial culture was spotted onto the center of LB agar plates containing 0.3% agar. Each treatment had six replicates. The diameter of colonies was measured after incubating the plates in a 30 °C incubator without moving or shaking for 48 h. During incubation, it was crucial that the plates were not disturbed or tilted. Due to bacterial motility, single colonies formed thin, translucent halos in the center of the plates, which were photographed and analyzed after independently repeating this experiment three times.

#### 4.3.4. Secretion of T6SS Effector Protein Hcp

The Hcp secretion of strains RS-1 and RS-2 was detected using the Enzyme-linked immunosorbent assay (ELISA) as described by Masum et al. [65]. Briefly, 1 mL of overnight and 30 °C bacterial culture (approximately OD_600_ = 0.6) was harvested by centrifugation at 4000× *g* for 10 min, and the supernatant was then filtered through a 0.22 μm filter. The microtiter plates were coated with 150 μL of filtered antigen diluted 10 times with coating buffer and incubated overnight at 4 °C. The plates were blocked with 175 μL/well of blocking buffer (phosphate-buffered saline (PBS), 10 mM CaCl_2_, 1% bovine serum albumin (BSA), 0.05% Tween 20) for 1 h at 37 °C and then washed with washing buffer. Next, 150 μL anti-Hcp antibody (1:5000 dilution) was added into each well of the microtiter plate and incubated at 37 °C for 1 h. After washing three times with PBST, the polyclonal antibody (AP-conjugated Goat Anti-Rabbit IgG at a 1:5000 dilution from Sangon Biotech (Shanghai, China) was used. After washing three times with PBST, 100 μL of the substrate PNPP (Disodium 4-Nitrophenyl Phosphate Hexahydrate) solution was added into each well, and the plate was placed at room temperature for 20 min; then, the OD_450_ was measured_._ Each treatment was performed in triplicate and repeated three times.

#### 4.3.5. Pathogenicity

The pathogenicity of strains RS-1 and RS-2 was determined based on the method by Li et al. [2], which was carried out by planting bacterized seed rice seeds in perlite. Bacterized rice seeds were prepared by soaking healthy rice seeds in overnight bacterial suspension with an OD_600_ of 1.0 (approximately 10^9^ CFU/mL) and shaking at 250 rpm/min at 30 °C, for about 2 h. After incubating the bacterized seeds on moist Petri dishes with double-layer filter paper at 30 °C with 16 h of light per day and 80% humidity, the germinated seeds with uniform shoot lengths were sowed into sterilized perlite, with 15 seeds per pot. After 10 d of sowing, the disease symptoms were observed, and the plant height was measured. Each treatment was replicated, and the experiment was repeated three times.

### 4.4. Identification of Genes Involved in Phage Infection

#### 4.4.1. Comparative Genomics Analysis

The mechanism for resistance and sensitivity of phage AP1 to *A. oryzae* was analyzed by sequencing and comparing the genomes of strains RS-1 and RS-2. Bacterial genomes were extracted using the Genomic DNA Purification Kit according to the instructions (B518255, Sangon Biotech Co., Ltd., Shanghai, China) and sequenced by Hangzhou Guhe Biotechnology Co., Ltd. (Hangzhou, China) using third-generation sequencing technology. Genome alignment was performed by Mauve software (Version 2.4.0, Sydney, Australia), while protein classification was carried out by using Blastp and the database of Clusters of Orthologous Genes (COGs).

#### 4.4.2. Mutation and Complementation of Unique Genes

Using the genome of the wild-type strain as the template, mutation and complementation of unique genes in strain RS-2 was carried out as described earlier [66]. Genes were functionally inactivated using insertional mutagenesis. Briefly, following the amplification of a 300–500 bp fragment in the middle of the target gene, the DNA fragment was digested with a restriction enzyme (*Eco*RI and *Bam*HI) and ligated into a pJP5603 suicide plasmid. The validated recombinant suicide plasmid was transformed into a wild-type strain by electric shock and spread on an LB agar medium containing 50 μg/mL kanamycin. The mutant strain was identified by PCR amplification and sequence analysis of 16S rDNA. For the construction of the complement strain, the target gene with about a 500 bp upstream fragment was amplified and cloned into a pRADK plasmid by using the Sosoo Cloning Kit (Tsingke Biotechnology Co., Ltd., Beijing, China). The recombinant plasmid was transformed into a mutant strain by electric shock. Following the screening of 50 μg/mL kanamycin and 10 μg/mL chloromycetin resistance, the complementary strain was verified by PCR amplification and sequence analysis of 16S rDNA. Primers used to make the unique gene mutants and their complementation are listed in Table 4.

### 4.5. Identification of LPS Receptor

The LPS receptors of susceptible strain RS-2 were determined as described previously by Kiljunen et al. [67] by using the phage adsorption assay. This was carried out by treating the overnight bacterial culture with sodium acetate (50 mM, pH 5.2) or sodium acetate containing 100 mM periodate at room temperature in the dark for 2 h. None was used as a non-adsorbing control. RS-2 treated by ddH_2_O was used as a positive control for complete adsorption. The basic procedure was mentioned above; specifically, the concentration of phage AP1 used here was 10^7^ PFU/mL. Each treatment was performed in triplicate, and the experiment was repeated three times.

### 4.6. LPS Binding of Phage Structural Proteins

The complete sequences of six putative phage structural proteins, gp47, gp48, gp65, gp66, gp67, and gp69, were cloned into the plasmid pGEX-6 containing a soluble GST-tagged protein and then transformed into the *E. coli* expression strain BL21 (DE3) for IPTG-induced expression. The purified protein was bound to BeyoGold™ GST-tag Purification Resin (Beyotime, Shanghai, China). The mixture was added to the column, and the bottom cap was opened to allow the liquid inside the column to flow out under gravity. The column was washed 5 times with 0.5–1 mL of lysis buffer each time. Then, 0.5 mL of lipopolysaccharide (LPS) extracted from RS-2 was slowly added to the column. The liquid inside the column was allowed to flow out under gravity. The column was washed 5 times with 0.5–1 mL of lysis buffer each time. The target protein was eluted 6–10 times with 0.5 mL of elution buffer each time. Each eluate was collected into separate centrifuge tubes. The collected eluates contained the LPS complexes bound to the protein. The concentration of LPS was measured using the anthrone-sulfuric acid method. The sugar content bound to the GST protein, used as a negative control, was subtracted from each sample. The binding proportion to LPS was calculated based on the initial amount of LPS before loading.

### 4.7. Receptor Proteins Involved in Strain-Specific Infection

#### 4.7.1. Determination by Protein Pull-Down

This experiment followed similar basic steps to the LPS binding assay. Purified GST-tagged proteins were used as bait in the protein pull-down assay. The entire protein of RS-2 was passed through an immunoadsorption column, resulting in protein complexes, which were denatured and subjected to SDS-PAGE electrophoresis using a 4–20% gradient gel. After Coomassie brilliant blue staining, the bands were observed. Differential bands were excised, enzymatically digested, and then analyzed by mass spectrometry (Orbitrap Elite MS, Thermo Fisher Scientific Inc., Waltham, MA, USA).

#### 4.7.2. Determination by Bacterial Two-Hybrid Assay

Protein–protein interactions in vivo were determined by using a bacterial two-hybrid experiment, which was carried out as described by Chen et al. [58]. Firstly, primers were designed to PCR clone the entire gene sequence of RS-2. After double digestion, the fragments were ligated into the pKNT25 vector and transformed into *Escherichia coli* DH5α to obtain recombinant plasmids resistant to kanamycin (Km). Using the same method, gene sequences from AP1 were cloned into the pCH363 vector to obtain recombinant plasmids resistant to ampicillin. Furthermore, 5 μL of each recombinant plasmid were mixed and co-transformed into *Escherichia coli* BTH101 competent cells by heat shock transformation, with a heat shock time of 90 s at 42 °C. The transformed *E. coli* was then spread on LB plates containing both kanamycin (50 μg/mL) and ampicillin (100 μg/mL) and incubated overnight at 37 °C. Single colonies containing both recombinant plasmids were selected and transferred into 5 mL of fresh LB medium. The cultures were shaken at 37 °C and 200 rpm/min overnight; then, 5 μL of bacterial culture was spotted carefully onto LB solid plates containing 40 μg/mL X-Gal, 500 μM IPTG, 100 μg/mL ampicillin, and 50 μg/mL kanamycin and then incubated in the dark at 23 °C for 48 h. After incubation, the plates were photographed and analyzed.

## 5. Conclusions

In conclusion, this study revealed the underlying mechanism of differential interaction between phage AP1 and the rice bacterial brown stripe pathogen by comparing phenotypic and molecular characteristics of the phage-resistant strain RS-1 and the sensitive strain RS-2. Despite similarities in cell growth, biofilm formation, motility, and Hcp production, the strains exhibited distinct differences in pathogenicity and phage susceptibility. Host proteins interacting with the structural proteins of phage AP1 were identified through prokaryotic expression, LPS binding assays, pull-down experiments, and bacterial two-hybrid analysis. Lastly, host-specific OPS synthesis gene clusters were found to be related to phage adsorption, resulting in phage-specific infection following genomic analysis and comparison of both bacterial strains, as well as gene mutation and complementation methods. Overall, these findings demonstrate that LPS serves as the receptor for AP1 on the host cell surface, and a unique gene cluster involved in LPS precursor biosynthesis influences the adsorption efficiency of AP1. This study provides valuable insights for the optimization and application of phage therapy against *A. oryzae* and related pathogens.

## Figures and Tables

**Table 1:** Proteins in RS-2 co-eluted with phage structural protein gp66.

Protein	PSMs	Function
Orf2086	7	Glycosyl transferase
Orf1561	6	Transmembrane protein RcnB
Orf2292	3	T6SS ClpB
Orf2286	3	T6SS ImpB

PSM: Peptide-spectrum match.

**Table 2:** Genome information of strains RS-1 and RS-2.

Attribute	RS-1		RS-2	
	Value	% of Total	Value	% of Total
Genome size (bp)	5,580,732	100	5,757,931	100
DNA coding (bp)	4,978,677	89.21	5,138,955	89.25
DNA G + C (bp)	3,830,703	68.64	3,949,796	68.60
DNA scaffolds	1	100	1	100
Total genes	5070	100	5230	100
Protein-coding genes	4992	98.46	5155	98.57
RNA genes	78	1.54	75	1.43
Genes with function prediction	3966	79.45	4054	78.64
Genes assigned to COGs	3439	67.83	3496	66.85
Genes with Pfam domains	4039	79.66	4152	79.39
Genes with signal peptides	452	8.92	462	8.83
Genes with transmembrane helices	1120	22.09	1149	21.97
Genes with genome islands	185	3.65	195	3.73
Genes with virulence	1686	33.25	1728	33.04
Genes with secondary metabolism	1069	21.08	1113	21.28
Genes with antibiotic resistance	280	5.52	288	5.51

**Table 3:** List of plasmids and bacterial strains used in this study.

Name	Relevant Characteristics	Reference
*Acidovorax oryzae*		
RS-1	Amp^R^; the pathogen of bacterial brown stripe of rice, isolated from diseased rice from Zhejiang province in China. AP1-resistant strain.	Lab collection
RS-2	Amp^R^; the pathogen of bacterial brown stripe of rice, isolated from diseased rice from Zhejiang province in China. AP1-sensitive strain.	Lab collection
Unique gene mutants	Amp^R^, Km^R^; the unique gene mutants of *Acidovorax oryzae* RS-2.	This study
Unique gene complementary mutants	Amp^R^, Km^R^, Chl^R^; the unique gene complementary mutants of *Acidovorax oryzae* RS-2.	This study
*Escherichia coli*		
DH5α	F-Φ80d lacZΔM15Δ (lacZYA-argF) U169 recA1 endA1, hsdR17(rk^–^, mk^+^) phoAsupE44 λ-thi-1 gyrA96 relA1.	Invitrogen
S17-1 λ *pir*	λ Lysogenic S17-1 derivative producing π protein for replication of plasmids carrying oriR6K; recA pro hsdR RP4-2-Tc::Mu-Km::Tn7 λ–pir.	[56]
BTH101	Host for overexpressing proteins in bacterial two-hybrid test.	[57]
BL21(DE3)	Host for overexpressing proteins driven by T7 promoter.	Invitrogen
Plasmids		
pJP5603	Km^R^; R6K-based suicide vector; requires the pir-encoded π protein for replication.	[56]
pGEX-6p-1	Amp^R^; expression vector with GST label.	Promega
pGEX-6p-1-*gp47*	Amp^R^; recombinant expression vector with GST label with a *gp47*.	This study
pGEX-6p-1-*gp48*	Amp^R^; recombinant expression vector with GST label with a *gp48*.	This study
pGEX-6p-1-*gp65*	Amp^R^; recombinant expression vector with GST label with a *gp65*.	This study
pGEX-6p-1-*gp66*	Amp^R^; recombinant expression vector with GST label with a *gp66*.	This study
pGEX-6p-1-*gp67*	Amp^R^; recombinant expression vector with GST label with a *gp67*.	This study
pGEX-6p-1-*gp69*	Amp^R^; recombinant expression vector with GST label with a *gp69*.	This study
pCH363	Amp^R^; expression vector for bacterial two-hybrid test.	[58]
pKNT25	Km^R^; expression vector for bacterial two-hybrid test.	[58]
pCH-*66*	Amp^R^; recombinant expression vector for bacterial two-hybrid test.	This study
pKNT-*GT*	Km^R^; recombinant expression vector for bacterial two-hybrid test.	This study
pKNT-*RcnB*	Km^R^; recombinant expression vector for bacterial two-hybrid test.	This study
pKNT-*ClpB*	Km^R^; recombinant expression vector for bacterial two-hybrid test.	This study

**Table 4:** List of oligo nucleotide PCR primers used in this study.

Name	Sequence (5′-3′)	Usage
*Ao*	GACCAGCCACACTGGGAC/CTGCCGTACTCCAGCGAT	*A. oryzae* screen
*16S rRNA*	AGAGTTTGATCCTGGCTCAG/GGCTACCTTGTTACGACTTC	16S rDNA
*gp47*	ATATAGGATCCATGGCCCTGCGCAAGAACTCCTC/TGTCAGTCGACTCAGCCTTGCGCGAAGGCG	Prokaryotic expression of the corresponding proteins
*gp48*	ATAGGATCCATGAAGTTCTACGCCCCCACCG/TGTCAGTCGACTCAGCCGTTCACGTCTTCGAAG	
*gp65*	ATATAGGATCCGTGCCGGGCAAAGTGCTCG/TGTCAGTCGACTCATGGTCCACGCGTCACCT	
*gp66*	CGGGATCCGTGGACCATGACGTGCTTTTCG/TGTCAGTCGACCTATGCGAGGTTGATGGGTGGTTG	
*gp67*	CGGGATCCATGGCCGCAAAATCTTTCATTCGC/TGTCAGTCGACTTATGCCAGTACCACCGGCAG	
*gp69*	CGGGATCCATGAGCCTTCGCTACCAACTGAC/TGTCAGTCGACTCAGTCCCTATGCACGGTGGAC	
*gp66 B2H*	CGGGATCCGTGGACCATGACGTGCTTTTCG/CGGAATTCCTATGCGAGGTTGATGGGTGGTTG	Full sequence of the corresponding proteins for B2H
*GT B2H*	CGGGATCCGTGAACCGCGCCCGCATCT/CGGAATTCCTACAGCCCGAAGAAGGGGCG	
*RcnB B2H*	CGGGATCCATGATCCGTACAGACTCCTCTTGCG/CGGAATTCCTACTGGAAGACGATCTGCGCG	
*ClpB B2H*	GCTCTAGAATGTCTGAAATCAGCCGGCAAGC/CGGAATTCTCAGGCCTCCTCGTACTCGATG	
*ImpB B2H*	CGGGATCCATGGTGACCTTAAGCAAGACCGG/CGGAATTCTCACTGAGCGTCGTCGGTCTT	
*1713*	CGCGGATCCGCAGGGCGGCAAAGAT/CCGGAATTCCGTTCACCAGGGCGATA	Primers used for the creation of mutants
*1714*	CGGGATCCTGGGCTGCGATGTGA/GGAATTCGCTTCTTGCCTTTGACG	
*1715*	CGGGATCCCAGTGCCGTATTCACCAA/CCGGAATTCCTTCGCCATTTCCAGTC	
*1716*	CGCGGATCCCGAAATCGCAGCACG/CCGGAATTCTGAGGAACGCAATGACC	
*1717*	CGGGATCCCAACACTACGATGCGAACA/CCGGAATTCAAGCAGGCTGGAAACC	
*1717.1*	CGGGATCCCCAGTAAAGGCGGTTCT/CGGAATTCGGGCATCCCGATAAAG	
*1718*	CGCGGATCCCGCTCCTGGACGATGT/CCGGAATTCCCGCAGATGCACCTATT	
*1718.1*	CGGGATCCCAAGATGCGTGGCGTGAA/CGGAATTCGCTCGTCGGAAGGAACCC	
*1718.2*	CGGGATCCGGGGTATGAGTGGGAGTTGC/CGGAATTCATTTATTGCTGCCTGGGTTTT	
*1718.3*	CGGGATCCATTGCCGTTCCGTATCGC/CGGAATTCAACTGACATTGCCTGGTATGCT	
*1719*	CGGGATCCGGACCTGATGGAGACGG/CCGGAATTCCGGGCACAGAACACCT	
*1720*	CGCGGATCCAGGTTCCTCACGGTTTCT/CCGGAATTCCGCTCGCAGATTTCG	
*1721*	CGGGATCCATGTCCTTCCTCGTCTCG/CCGGAATTCCAATCGCACGCTGTCC	
*1722*	CGCGGATCCTTCTCAGCACCCAATCAT/CCGGAATTCAGGTCCGTGGCAAATC	
*1723*	CGCGGATCCGCATTACCTGCTCTATGACTT/CCGGAATTCGGCGGATACACCACTTCT	
*pRADK-1714*	TGCCATGGTACCCGGGAGCTCGGGAGTTCGAATCCTGGGG/CGCGTCTGCATGTGGAAGCTTTCAACGAACGAAGGGATGCC	Primers used for the creation of complementary strains
*pRADK-1715*	TGCCATGGTACCCGGGAGCTCCCCGACGACGTCAAAGGC/CGCGTCTGCATGTGGAAGCTTTCAAATACGTTGGACCCGGC	
*pRADK-1716*	TGCCATGGTACCCGGGAGCTCCGCCGGACCGTCGTCCGC/CGCGTCTGCATGTGGAAGCTTTCATTTTTCCAATGCCATTGC	
*pRADK-1717*	TGCCATGGTACCCGGGAGCTCTCCCGAAGTCGTTCGGCC/CGCGTCTGCATGTGGAAGCTTTCAGTCAGCACCCATGATTTCC	
*pRADK-1718*	TGCCATGGTACCCGGGAGCTCGGTCACAGACCCGTTGCCA/CGCGTCTGCATGTGGAAGCTTTCAAATAGTCCATTCCACGGGA	

All primers were designed in this study.

## Data Availability

All data supporting the conclusions of this article are included in this article. The genome sequences of strains RS-1 and strain RS-2 were deposited on NCBI with Accession nos. CP169238 and CP169239.

## References

[1] Kakar K.U., Nawaz Z., Cui Z., Almoneafy A.A., Zhu B., Xie G.L. (2014). Characterizing the Mode of Action of *Brevibacillus laterosporus* B4 for Control of Bacterial Brown Strip of Rice Caused by *A*. *avenae* subsp. *avenae* RS-1. World J. Microbiol. Biotechnol..

[2] Li B., Liu B., Yu R., Tao Z., Wang Y., Xie G., Li H., Sun G. (2011). Bacterial Brown Stripe of Rice in Soil-Less Culture System Caused by *Acidovorax avenae* subsp. *avenae* in China. J. Gen. Plant Pathol..

[3] Liu H., Yang C.L., Ge M.Y., Ibrahim M., Li B., Zhao W.J., Chen G.Y., Zhu B., Xie G.L. (2014). Regulatory Role of tetR Gene in a Novel Gene Cluster of *Acidovorax avenae* subsp. *avenae* RS-1 under Oxidative Stress. Front. Microbiol..

[4] Wang Y., Zhou Q., Li B., Liu B., Wu G., Ibrahim M., Xie G., Li H., Sun G. (2012). Differentiation in MALDI-TOF MS and FTIR Spectra between Two Closely Related Species *Acidovorax oryzae* and *Acidovorax citrulli*. BMC Microbiol..

[5] Yang C., Li B., Ge M., Zhou K., Wang Y., Luo J., Ibrahim M., Xie G., Sun G. (2014). Inhibitory Effect and Mode of Action of Chitosan Solution against Rice Bacterial Brown Stripe Pathogen *Acidovorax avenae* subsp. *avenae* RS-1. Carbohydr. Res..

[6] Miura T., Kusada H., Kamagata Y., Hanada S., Kimura N. (2013). Genome Sequence of the Multiple-β-Lactam-Antibiotic-Resistant Bacterium *Acidovorax* sp. Strain MR-S7. Genome Announc..

[7] Jia H.J., Jia P.P., Yin S., Bu L.K., Yang G., Pei D.-S. (2023). Engineering Bacteriophages for Enhanced Host Range and Efficacy: Insights from Bacteriophage-Bacteria Interactions. Front. Microbiol..

[8] Rakhuba D.V., Kolomiets E.I., Dey E.S., Novik G.I. (2010). Bacteriophage Receptors, Mechanisms of Phage Adsorption and Penetration into Host Cell. Pol. J. Microbiol..

[9] Stone E., Campbell K., Grant I., McAuliffe O. (2019). Understanding and Exploiting Phage–Host Interactions. Viruses.

[10] Geller B.L., Ivey R.G., Trempy J.E., Hettinger-Smith B. (1993). Cloning of a Chromosomal Gene Required for Phage Infection of *Lactococcus lactis* subsp. *lactis* C2. J. Bacteriol..

[11] Vegge C.S., Vogensen F.K., Mc Grath S., Neve H., Van Sinderen D., Brøndsted L. (2006). Identification of the Lower Baseplate Protein as the Antireceptor of the Temperate *Lactococcal* Bacteriophages TP901-1 and Tuc2009. J. Bacteriol..

[12] Andres D., Roske Y., Doering C., Heinemann U., Seckler R., Barbirz S. (2012). Tail Morphology Controls DNA Release in Two *Salmonella* Phages with One Lipopolysaccharide Receptor Recognition System. Mol. Microbiol..

[13] Barbirz S., Müller J.J., Uetrecht C., Clark A.J., Heinemann U., Seckler R. (2008). Crystal Structure of *Escherichia Coli* Phage HK620 Tailspike: Podoviral Tailspike Endoglycosidase Modules are Evolutionarily Related. Mol. Microbiol..

[14] Schwarzer D., Browning C., Stummeyer K., Oberbeck A., Mühlenhoff M., Gerardy-Schahn R., Leiman P.G. (2015). Structure and Biochemical Characterization of Bacteriophage Phi92 Endosialidase. Virology.

[15] Stummeyer K., Dickmanns A., Mühlenhoff M., Gerardy-Schahn R., Ficner R. (2005). Crystal Structure of the Polysialic Acid–Degrading Endosialidase of Bacteriophage K1F. Nat. Struct. Mol. Biol..

[16] Subramanian S., Dover J.A., Parent K.N., Doore S.M. (2022). Host Range Expansion of *Shigella* Phage Sf6 Evolves through Point Mutations in the Tailspike. J. Virol..

[17] Walter M., Fiedler C., Grassl R., Biebl M., Rachel R., Hermo-Parrado X.L., Llamas-Saiz A.L., Seckler R., Miller S., Van Raaij M.J. (2008). Structure of the Receptor-Binding Protein of Bacteriophage Det7: A Podoviral Tail Spike in a *Myovirus*. J. Virol..

[18] Witte S., Zinsli L.V., Gonzalez-Serrano R., Matter C.I., Loessner M.J., Van Mierlo J.T., Dunne M. (2021). Structural and Functional Characterization of the Receptor Binding Proteins of *Escherichia coli* O157 Phages EP75 and EP335. Comput. Struct. Biotechnol. J..

[19] Li H., Marceau M., Yang T., Liao T., Tang X., Hu R., Xie Y., Tang H., Tay A., Shi Y. (2019). East-Asian *Helicobacter pylori* Strains Synthesize Heptan-Deficient Lipopolysaccharide. PLoS Genet..

[20] Tang X., Wang P., Shen Y., Song X., Benghezal M., Marshall B.J., Tang H., Li H. (2023). Lipopolysaccharide O-Antigen Profiles of *Helicobacter pylori* Strains from Southwest China. BMC Microbiol..

[21] Gao S., Jin W., Quan Y., Li Y., Shen Y., Yuan S., Yi L., Wang Y., Wang Y. (2024). Bacterial Capsules: Occurrence, Mechanism, and Function. npj Biofilms. Microbiomes..

[22] Berry M.C., McGhee G.C., Zhao Y., Sundin G.W. (2009). Effect of a waaL Mutation on Lipopolysaccharide Composition, Oxidative Stress Survival, and Virulence in *Erwinia amylovora*. FEMS Microbiol. Lett..

[23] Clifford J.C., Rapicavoli J.N., Roper M.C. (2013). A Rhamnose-Rich O-Antigen Mediates Adhesion, Virulence, and Host Colonization for the Xylem-Limited Phytopathogen *Xylella fastidiosa*. Mol. Plant Micobe.

[24] Li C.H., Wang K.C., Hong Y.H., Chu T.H., Chu Y.J., Chou I.C., Lu D.K., Chen C.Y., Yang W.C., Lin Y.M. (2014). Roles of Different Forms of Lipopolysaccharides in *Ralstonia solanacearum* Pathogenesis. Mol. Plant Micobe..

[25] Petrocelli S., Tondo M.L., Daurelio L.D., Orellano E.G. (2012). Modifications of *Xanthomonas axonopodis* pv. *citri* Lipopolysaccharide Affect the Basal Response and the Virulence Process during Citrus Canker. PLoS ONE.

[26] Dy R.L., Richter C., Salmond G.P.C., Fineran P.C. (2014). Remarkable Mechanisms in Microbes to Resist Phage Infections. Annu. Rev. Virol..

[27] Rostøl J.T., Marraffini L. (2019). (Ph)Ighting Phages: How Bacteria Resist Their Parasites. Cell Host Microbe.

[28] Fortuna M.A., Barbour M.A., Zaman L., Hall A.R., Buckling A., Bascompte J. (2019). Coevolutionary Dynamics Shape the Structure of Bacteria-phage Infection Networks. Evolution.

[29] Koskella B., Brockhurst M.A. (2014). Bacteria–Phage Coevolution as a Driver of Ecological and Evolutionary Processes in Microbial Communities. FEMS Microbiol. Rev..

[30] Liu M., Tian Y., Zaki H.E.M., Ahmed T., Yao R., Yan C., Leptihn S., Loh B., Shahid M.S., Wang F. (2022). Phage Resistance Reduced the Pathogenicity of *Xanthomonas oryzae* pv. *oryzae* on Rice. Viruses.

[31] Zhang M., Wang Y., Chen J., Hong X., Xu X., Wu Z., Ahmed T., Loh B., Leptihn S., Hassan S. (2022). Identification and Characterization of a New Type of Holin-Endolysin Lysis Cassette in *Acidovorax oryzae* Phage AP1. Viruses.

[32] Frampton R.A., Taylor C., Holguín Moreno A.V., Visnovsky S.B., Petty N.K., Pitman A.R., Fineran P.C. (2014). Identification of Bacteriophages for Biocontrol of the Kiwifruit Canker Phytopathogen *Pseudomonas syringae* pv. *actinidiae*. Appl. Environ. Microbiol..

[33] Gill J.J., Svircev A.M., Smith R., Castle A.J. (2003). Bacteriophages of *Erwinia amylovora*. Appl. Environ. Microbiol..

[34] Bhunchoth A., Phironrit N., Leksomboon C., Chatchawankanphanich O., Kotera S., Narulita E., Kawasaki T., Fujie M., Yamada T. (2015). Isolation of *Ralstonia solanacearum*-infecting Bacteriophages from Tomato Fields in Chiang Mai, Thailand, and Their Experimental Use as Biocontrol Agents. J. Appl. Microbiol..

[35] Dunne M., Prokhorov N.S., Loessner M.J., Leiman P.G. (2021). Reprogramming Bacteriophage Host Range: Design Principles and Strategies for Engineering Receptor Binding Proteins. Curr. Opin. Biotechnol..

[36] Miller E.S., Kutter E., Mosig G., Arisaka F., Kunisawa T., Rüger W. (2003). Bacteriophage T4 Genome. Microbiol. Mol. Biol. Rev..

[37] Casjens S.R., Hendrix R.W. (2015). Bacteriophage Lambda: Early Pioneer and Still Relevant. Virology.

[38] Maffei E., Shaidullina A., Burkolter M., Heyer Y., Estermann F., Druelle V., Sauer P., Willi L., Michaelis S., Hilbi H. (2021). Systematic Exploration of Escherichia Coli Phage–Host Interactions with the BASEL Phage Collection. PLoS Biol..

[39] De Jonge P.A., Nobrega F.L., Brouns S.J.J., Dutilh B.E. (2019). Molecular and Evolutionary Determinants of Bacteriophage Host Range. Trends Microbiol..

[40] Bertozzi Silva J., Storms Z., Sauvageau D. (2016). Host Receptors for Bacteriophage Adsorption. FEMS Microbiol. Lett..

[41] Ha E., Chun J., Kim M., Ryu S. (2019). Capsular Polysaccharide Is a Receptor of a *Clostridium perfringens* Bacteriophage CPS1. Viruses.

[42] Letarov A.V., Kulikov E.E. (2017). Adsorption of Bacteriophages on Bacterial Cells. Biochemistry.

[43] Lindberg A.A. (1973). Bacteriophage Receptors. Annu. Rev. Microbiol..

[44] Kiljunen S., Hakala K., Pinta E., Huttunen S., Pluta P., Gador A., Lönnberg H., Skurnik M. (2005). Yersiniophage ϕR1-37 Is a Tailed Bacteriophage Having a 270 Kb DNA Genome with Thymidine Replaced by Deoxyuridine. Microbiology.

[45] Pinta E., Duda K.A., Hanuszkiewicz A., Salminen T.A., Bengoechea J.A., Hyytiäinen H., Lindner B., Radziejewska-Lebrecht J., Holst O., Skurnik M. (2010). Characterization of the Six Glycosyltransferases Involved in the Biosynthesis of *Yersinia enterocolitica* Serotype O:3 Lipopolysaccharide Outer Core. J. Biol. Chem..

[46] Zhang M., Qian J., Xu X., Ahmed T., Yang Y., Yan C., Elsharkawy M.M., Hassan M.M., Alorabi J.A., Chen J. (2022). Resistance of *Xanthomonas oryzae* pv. *oryzae* to Lytic Phage X2 by Spontaneous Mutation of Lipopolysaccharide Synthesis-Related Glycosyltransferase. Viruses.

[47] Xu J., Zhang J., Lu X., Liang W., Zhang L., Kan B. (2013). O Antigen is the Receptor of *Vibrio cholerae* Serogroup O1 El Tor Typing Phage VP4. J. Bacteriol..

[48] Burrows L.L., Pigeon K.E., Lam J.S. (2000). *Pseudomonas aeruginosa* B-Band Lipopolysaccharide Genes *wbpA* and *wbpI* and Their *Escherichia coli* Homologues *wecC* and *wecB* are Not Functionally Interchangeable. FEMS Microbiol. Lett..

[49] Wenzel C.Q., Daniels C., Keates R.A.B., Brewer D., Lam J.S. (2005). Evidence that WbpD is an *N*-acetyltransferase Belonging to the Hexapeptide Acyltransferase Superfamily and an Important Protein for O-antigen Biosynthesis in *Pseudomonas aeruginosa* PAO1. Mol. Microbiol..

[50] Westman E.L., Preston A., Field R.A., Lam J.S. (2008). Biosynthesis of a Rare Di-N-Acetylated Sugar in the Lipopolysaccharides of Both *Pseudomonas aeruginosa* and *Bordetella pertussis* Occurs via an Identical Scheme despite Different Gene Clusters. J. Bacteriol..

[51] Capotosti F., Guernier S., Lammers F., Waridel P., Cai Y., Jin J., Conaway J.W., Conaway R.C., Herr W. (2011). O-GlcNAc Transferase Catalyzes Site-Specific Proteolysis of HCF-1. Cell.

[52] Martinez-Fleites C., Macauley M.S., He Y., Shen D.L., Vocadlo D.J., Davies G.J. (2008). Structure of an O-GlcNAc Transferase Homolog Provides Insight into Intracellular Glycosylation. Nat. Struct. Mol. Biol..

[53] Pei T., Yan M., Li T., Li X., Yin Y., Cui M., Fang Y., Liu J., Kong Y., Xu P. (2022). Characterization of UDP-Glycosyltransferase Family Members Reveals How Major Flavonoid Glycoside Accumulates in the Roots of *Scutellaria baicalensis*. BMC Genom..

[54] Thoden J.B., Holden H.M. (2011). Biochemical and Structural Characterization of WlbA from *Bordetella pertussis* and *Chromobacterium violaceum*: Enzymes Required for the Biosynthesis of 2,3-Diacetamido-2,3-Dideoxy-d-Mannuronic Acid. Biochemistry.

[55] Ge X., Wang J. (2024). Structural Mechanism of Bacteriophage Lambda Tail’s Interaction with the Bacterial Receptor. Nat. Commun..

[56] Xie G.L., Zhang G.Q., Liu H., Lou M.M., Tian W.X., Li B., Zhou X.P., Zhu B., Jin G.L. (2011). Genome Sequence of the Rice-Pathogenic Bacterium *Acidovorax avenae* subsp. *avenae* RS-1. J. Bacteriol..

[57] Simon R., Priefer U., Pühler A. (1983). A Broad Host Range Mobilization System for In Vivo Genetic Engineering: Transposon Mutagenesis in Gram Negative Bacteria. Nat. Biotechnol..

[58] Chen Y., Chai Y., Guo J., Losick R. (2012). Evidence for Cyclic Di-GMP-Mediated Signaling in *Bacillus subtilis*. J. Bacteriol..

[59] Ogunyemi S.O., Chen J., Zhang M., Wang L., Masum M.M.I., Yan C., An Q., Li B., Chen J. (2019). Identification and Characterization of Five New OP2-Related *Myoviridae* Bacteriophages Infecting Different Strains of *Xanthomonas oryzae* pv. *oryzae*. J. Plant Pathol..

[60] Springer K., Reuter S., Knüpfer M., Schmauder L., Sänger P.-A., Felsl A., Fuchs T.M. (2018). Activity of a Holin-Endolysin System in the Insecticidal Pathogenicity Island of *Yersinia enterocolitica*. J. Bacteriol..

[61] Zhao X., Cui Y., Yan Y., Du Z., Tan Y., Yang H., Bi Y., Zhang P., Zhou L., Zhou D. (2013). Outer Membrane Proteins Ail and OmpF of *Yersinia pestis* are Involved in the Adsorption of T7-Related Bacteriophage Yep-Phi. J. Virol..

[62] Luo J., Qiu W., Chen L., Anjum S., Yu M., Shan C., Ilyas M., Li B., Wang Y., Sun G. (2015). Identification of Pathogenicity-Related Genes in Biofilm-Defective *Acidovorax citrulli* by Transposon Tn5 Mutagenesis. Int. J. Mol. Sci..

[63] Coenye T., Peeters E., Nelis H.J. (2007). Biofilm Formation by Propionibacterium Acnes is Associated with Increased Resistance to Antimicrobial Agents and Increased Production of Putative Virulence Factors. Res. Microbiol..

[64] Bahar O., Goffer T., Burdman S. (2009). Type IV Pili are Required for Virulence, Twitching Motility, and Biofilm Formation of *Acidovorax avenae* subsp. *citrulli*. Mol. Plant. Micobe..

[65] Masum M., Yang Y., Li B., Olaitan O., Chen J., Zhang Y., Fang Y., Qiu W., Wang Y., Sun G. (2017). Role of the Genes of Type VI Secretion System in Virulence of Rice Bacterial Brown Stripe Pathogen *Acidovorax avenae* subsp. *avenae* Strain RS-2. Int. J. Mol. Sci..

[66] Liu H., Tian W.X., Ibrahim M., Li B., Zhang G.Q., Zhu B., Xie G.L. (2012). Characterization of *pilP*, a Gene Required for Twitching Motility, Pathogenicity, and Biofilm Formation of *Acidovorax avenae* subsp. *avenae* RS-1. Eur. J. Plant Pathol..

[67] Kiljunen S., Datta N., Dentovskaya S.V., Anisimov A.P., Knirel Y.A., Bengoechea J.A., Holst O., Skurnik M. (2011). Identification of the Lipopolysaccharide Core of *Yersinia pestis* and *Yersinia pseudotuberculosis* as the Receptor for Bacteriophage φA1122. J. Bacteriol..

